# Effect of Waste Metal and Chamotte Fillers on the Thermal and Mechanical Properties of Geopolymer Composites for Energy Storage Applications

**DOI:** 10.3390/ma18163853

**Published:** 2025-08-17

**Authors:** Aleš Soukup, Mohammadtaghi Vakili, Pavlína Hájková

**Affiliations:** 1 Department of Material Science, Faculty of Mechanical Engineering, Technical University of Liberec, Studentská 1402/2, 461 17 Liberec, Czech Republic; pavlina.hajek@seznam.cz; 2 ORLEN UNIPETROL RPA s.r.o., Záluží 1, 436 70 Litvínov, Czech Republic

**Keywords:** geopolymer composite, metakaolinite, waste steel chips, thermal energy storage

## Abstract

This study investigates the effects of varying filler content on the thermal and mechanical performance of metakaolinite-based geopolymer composites designed for thermal energy storage applications. The composites were formulated using a geopolymer binder, combined with a thermally stable filler (ground chamotte) and a thermal energy storage filler (waste steel chips) in different proportions. Chamotte content within the binder matrix (binder + chamotte) ranged from 20 to 40 wt.%, while steel chip content varied from 0 to 40 wt.% of the total composite mass. The thermal properties of the composites were evaluated at room temperature and compared with conventional reference materials, including Ultraboard, chamotte brick, and magnetite brick. Mechanical performance, specifically flexural and compressive strength, was evaluated at room temperature and after exposure to elevated temperatures (800 and 1100 °C), followed by two cooling regimes, slow furnace cooling and rapid water quenching. Microstructural characterization via optical microscopy was used to examine filler dispersion and matrix–filler interactions. The results showed that the thermal effusivity of the optimized composites exceeded that of chamotte brick by more than 50%. The highest flexural (12.68 MPa) and compressive (86.18 MPa) strengths were achieved in the composite containing 20 wt.% steel chips, prior to thermal exposure. Microstructural observations revealed the diverse geometry of the steel chips and arrangement of the chamotte particles. These findings highlight the potential of incorporating metallic waste materials into geopolymer systems to develop multifunctional composites with improved thermal storage capacity and mechanical resilience.

## 1. Introduction

Geopolymers are inorganic polymeric materials synthesized by reacting aluminosilicate raw materials with alkaline activators. Over the past five decades, many research groups have investigated geopolymers and other alkali-activated aluminosilicates. This sustained interest is largely due to the significantly lower carbon footprint of geopolymer binders compared to traditional Portland cement-based ones, positioning them as a more environmentally friendly alternative. Their production also allows for the incorporation of industrial waste materials, thereby classifying them as sustainable binders [1,2,3,4]. Additional advantages of geopolymers include resistance to both high and low temperatures [5,6], as well as high mechanical strength and chemical resistance, particularly to organic solvents and acids [7]. However, one limitation is their high alkalinity, which introduces safety concerns due to corrosiveness, complicates handling and quality control, and may cause rapid setting, shrinkage, cracking, and reduced workability, which are factors that hinder large-scale practical use [8]. In industrial applications, they are primarily used as construction materials, providing an eco-friendly substitute for Portland cement [9]. They are also widely used in the fields of refractory materials and fire protection [10]. Geopolymers are promising for environmental applications such as thermal insulation and energy storage. Furthermore, geopolymers hold significant potential in the production of advanced non-flammable laminates for sectors such as the automotive and aerospace industries, where they can serve as lightweight, heat-resistant structural and protective materials [11]. In recent years, porous geopolymers have come to the forefront, playing a significant role in environmental applications as adsorption or separation media [12].

The synthesis of geopolymers occurs through geopolymerization, which is the partial dissolution of amorphous aluminosilicates in a highly alkaline environment, followed by polycondensation forming a three-dimensional (3D) network. Common precursors include natural or industrial materials rich in silicon dioxide (SiO_2_) and aluminum oxide (Al_2_O_3_), such as metakaolin [13], fly ash [14], blast furnace slag, or calcined claystone [15]. The main alkaline activators are sodium or potassium hydroxides and sodium or potassium silicate solutions, which influence both the reactivity of the system and the resulting microstructure [16,17]. Geopolymerization occurs in two main stages. Initially, the amorphous aluminosilicates partially dissolve in the alkaline solution, forming oligomeric silicate and aluminosilicate gels. This is followed by polycondensation, during which the oligomers bond to form a solid 3D polymer network [18,19].

Several factors determine the final properties of geopolymers, including the Si/Al ratio, the concentration of the activator, curing time and temperature, and the presence of fillers. These parameters influence both mechanical strength and thermal resistance [20,21,22]. By adjusting the composition of the base binder and the type and amount of added fillers, the properties of geopolymer materials can be tailored to meet specific application requirements [23,24,25]. This variability, along with the broad range of usable fillers, makes geopolymer binders suitable for diverse industrial and specialized applications.

When combined with various fillers, geopolymer binders form composites with enhanced mechanical and thermal performance, which are influenced by the type and distribution of the filler. Depending on the type of filler, geopolymer composites can be used at temperatures up to 1200 °C, withstand compressive loads exceeding 100 MPa, and resist a wide range of chemicals [6,7,10]. By combining the properties of the individual components of the composite materials, unique properties characterizing the composite material can be achieved. The high thermal conductivity and heat capacity of the waste metal improves heat storage, and the geopolymer binder transfers its property—high thermal stability—to the composite material. Geopolymer composites with metal particles exhibit excellent thermal conductivity, high bulk density, and strong thermal stability, showing great potential for thermal energy storage applications. Incorporating metallic waste into geopolymer matrices not only improves mechanical, thermal, and functional properties but also provides environmental and economic benefits. For example, Alserai et al. [26] studied the effect of adding iron waste on the mechanical properties of geopolymer and Portland cement composites and found that even small amounts of iron waste positively affected the strength of all mixtures tested. Similarly, Nongnuang et al. [27] incorporated iron dust from the steel industry into geopolymer composites and observed that, while the compressive strength was not significantly improved, the flexural strength more than doubled when 20% iron waste was added. Shaikh and Hosan [28] compared the performance of geopolymer and cement-based composites containing steel fibers at elevated temperatures (200, 400, 600, and 800 °C). Their results showed that sodium-based geopolymers maintained compressive and flexural strength up to 400 °C, whereas Portland cement composites experienced a nearly 30% reduction. All steel fiber composites also exhibited increased strength at ambient conditions, and the geopolymer-based composites showed fewer surface cracks after high-temperature exposure. Ranjbar et al. [29] studied the effect of adding steel and polypropylene fibers to geopolymer composites. While steel fibers improved strength, polypropylene fibers led to performance losses, likely due to poor interfacial bonding, as polypropylene has low surface energy and is hydrophobic.

Despite growing interest in the thermal performance of geopolymer composites, studies specifically examining their behavior when incorporating metallic particles remain limited. Prałat et al. [30] investigated the effect of metallic microparticles on the thermal conductivity and heat capacity of geopolymer composites, alongside other fillers such as chamotte and sand. They found no significant effects of iron on thermal conductivity in chamotte-based composites, while in sand-based composites (1:1 ratio), the iron addition reduced thermal conductivity. A key property of geopolymer composites is their thermal stability at elevated temperatures. For instance, Wang et al. [31] examined the effect of chromium dust addition (15%) on the properties of geopolymer composites after heating to 900, 1000, 1100, and 1200 °C. They observed significant increases in strength and thermal conductivity at 1100 °C, which was attributed to material densification and crystallization. Kuranlı et al. [32] evaluated geopolymer matrices reinforced with steel, polypropylene, and polyamide fibers after exposure to 300, 600, and 900 °C, as well as after cyclic freeze–thaw conditions. Their findings showed no significant changes in strength due to freeze–thaw cycles, but there was notable performance variation based on fiber type and temperature exposure. Most existing studies focus on metallic fillers in the form of powders or fibers. However, the use of metallic machining waste for enhancing the thermal storage potential of geopolymer composites remains largely unexplored, presenting a significant research opportunity.

Geopolymer composites offer improved performance at higher temperatures compared to conventional water-based thermal storage tanks and cement-based materials, which are typically limited to 400–500 °C [33]. They may also serve as alternatives to two-tank molten salt thermal energy storage systems, which, despite their operating temperatures up to 600 °C, require extensive thermal insulation due to their high solidification temperatures (120–220 °C) [34]. Geopolymer-based blocks could be used for heat storage in solar thermal systems, industrial processes, and other thermal applications. Colangelo et al. [35] confirmed the stability of geopolymer mixtures after repeated thermal cycling over 500 °C and down to 150 °C, with strength remaining high even at maximum temperatures.

This study investigates how fillers derived from waste metallic materials and ground chamotte affect the thermal and mechanical performance of geopolymer composites. The composite materials were prepared using a metakaolinite-based geopolymer binder with the following two filler types: metallic waste from cutting structural steel and thermally stable ground chamotte. Both thermal and mechanical properties of the composites were evaluated. The thermal performance of the prepared materials was compared to that of reference materials. To support comparative analysis, network graphs were constructed to visualize mechanical strength and thermal effusivity. The internal structure and filler distribution in the composites were also examined using optical microscopy.

## 2. Materials and Methods

### 2.1. Materials

The primary raw materials used for the preparation of thermal energy storage geopolymer composites were commercially available products. Mefisto L_05_, a metakaolinite-rich material obtained by calcining kaolinitic claystone in a rotary kiln at 750 °C (České lupkové závody, a.s., Nové Strašecí, Czech Republic), served as the primary aluminosilicate source. Potassium waterglass (Vodní sklo, a.s., Prague, Czech Republic) and potassium hydroxide flakes (G.R. grade, 91.7 wt.% KOH, Lach-Ner, s.r.o., Neratovice, Czech Republic) were employed as the alkaline activators. Ground chamotte A111 (České lupkové závody, a.s., Nové Strašecí, Czech Republic), with a particle size of 0–0.5 mm, was used as a thermally stable filler in the composite formulation.

**Figure 1:**
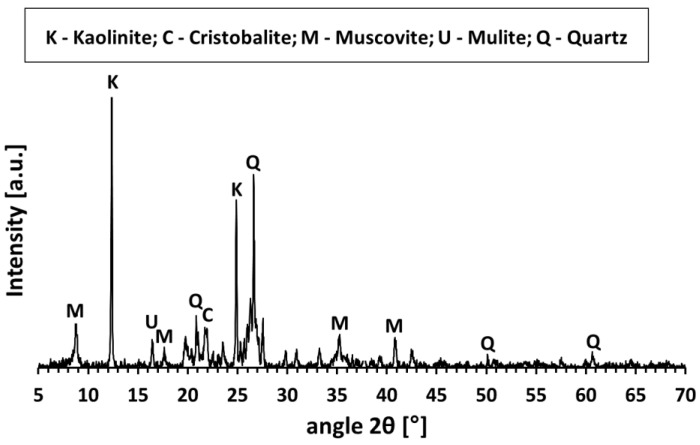
XRD patterns of aluminosilicate Mefisto L_05_.

**Figure 2:**
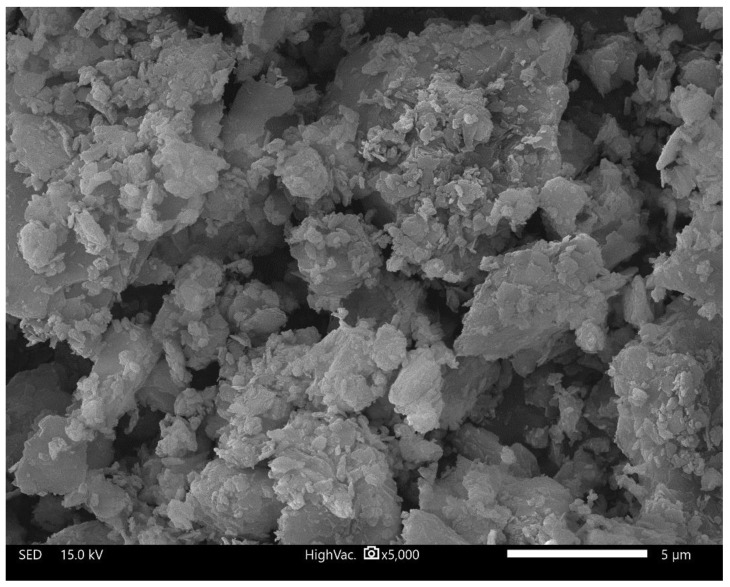
Morphology of the base material Mefisto L_05_.

**Figure 3:**
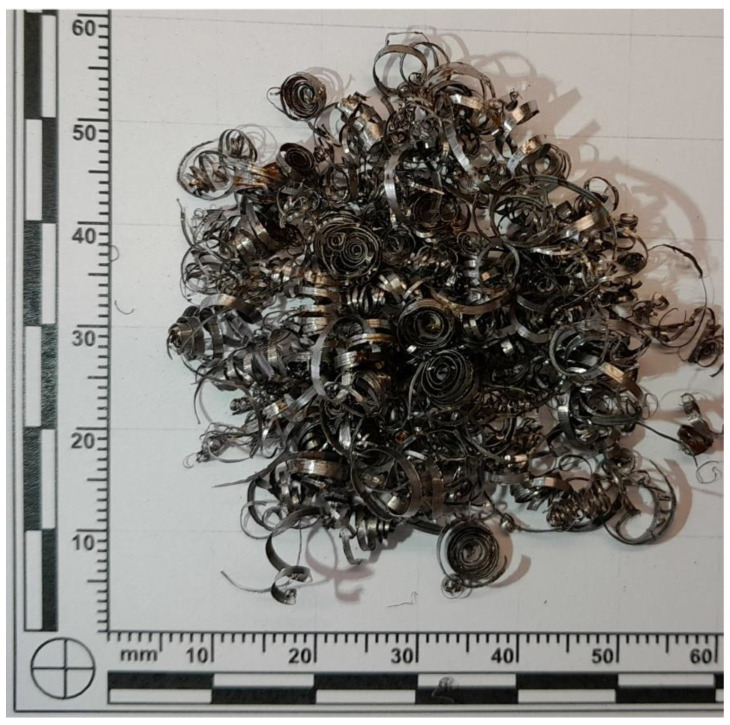
Waste steel shavings.

To introduce thermal energy storage capability, metallic waste generated during the cutting of structural steel was incorporated as a filler. This waste consisted of small steel chips, whose morphology depended on the type of structural steel and the specifications of the large-format circular saw used for cutting (Třinecké železárny, Sochorová válcovna, VZ Kladno). The filler represented a heterogeneous mixture of structural steel grades, typical of scrap metal sources. The phase composition of Mefisto L_05_ is presented in Figure 1. Table 1 presents the chemical composition of the primary raw materials, while Table 2 summarizes the physical properties of Mefisto L_05_ and the chamotte filler. Figure 2 provides a scanning electron microscope (SEM) image illustrating the morphology of Mefisto L_05_. A photograph of the metallic filler—steel chips—is shown in Figure 3.

To evaluate the thermal properties of the prepared composites, several commercially available reference materials were selected for comparison based on their thermal stability and refractoriness. These included one thermally insulating material and two designed for thermal energy storage. The insulating material, Ultraboard (M.E. SCHUPP Industriekeramik GmbH, Aachen, Germany), is a polycrystalline mullite/alumina board composed of inorganic fibers and organic binders. It withstands temperatures up to 1800 °C and is characterized by low thermal conductivity and minimal heat accumulation.

**Figure 4:**
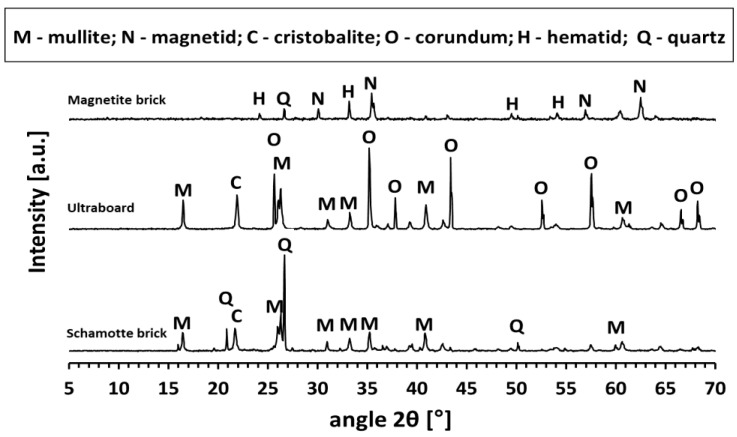
XRD patterns of reference materials.

The following two refractory bricks were used as thermal energy storage references: a stove chamotte brick (SIII-KP; RHI Magnetita Czech Republic a.s., Velké Opatovice, Czech Republic), resistant to repeated heating and cooling cycles with a maximum service temperature of 1100 °C, and a magnetite brick (MGT 50; RHI Magnesita Czech Republic a.s., Velké Opatovice, Czech Republic), notable for its high bulk density and thermal resistance up to 2200 °C. The phase compositions of these reference materials are presented in Figure 4, while their chemical compositions are listed in Table 3.

### 2.2. Analytical and Testing Methods

The phase composition of the aluminosilicate raw material Mefisto L_05_ was characterized using a BRUKER D8 Advance X-ray diffractometer (Bruker, Billerica, MA, USA) equipped with a BRUKER SSD 160 detector. The system operated with Cu-Kα radiation at 40 kV and 25 mA. Data were collected over a 2θ range of 5–70° using a step size of 0.02° and a dwell time of 1 s per step. The chemical composition of the solid materials (Mefisto L_05_ and chamotte) was determined via X-ray fluorescence (XRF) using a BRUKER S8 TIGER spectrometer (Bruker, Billerica, MA, USA). The chemical composition of the liquid potassium waterglass was analyzed using an OPTIMA 8000 inductively coupled plasma optical emission spectrometer (ICP-OES) (PerkinElmer, Waltham, MA, USA). In addition, the total content of alkali metals (K and Na), SiO_2_, and their molar ratios in the waterglass were assessed via standard acid–base titration, which is known to offer higher accuracy for more concentrated systems compared to alternative methods.

The specific gravity was measured using a Pycnomatic ATC Evo helium pycnometer (Microtrac, Osaka, Japan). Particle size distribution of the metakaolinite Mefisto L_05_ was determined with a Mastersizer 3000 laser diffraction particle size analyzer (Malvern Instruments, Malvern, UK), with ultrasonic dispersion applied to eliminate agglomerates prior to measurement. The Brunauer–Emmett–Teller (BET) surface area of Mefisto L_05_ was determined via nitrogen adsorption–desorption analysis using an Autosorb iQ instrument (Quantachrome Instruments, Boynton Beach, FL, USA).

Microstructural analysis of Mefisto L_05_ was performed using a JEOL JSM-IT500HR scanning electron microscope (JEOL, Tokyo, Japan). Samples were sputter-coated with a 5 nm layer of gold to ensure conductivity. Images were acquired in high-vacuum mode at an accelerating voltage of 15 kV and a magnification of 5000× (scale bar = 5 μm).

Thermal properties were evaluated using an ISOMET 2114 device (Applied Precision, Bratislava, Slovakia), which measures thermal conductivity (λ [W/m·K]), volumetric heat capacity (cp [J/m^3^·K]), and thermal diffusivity (a [m^2^/s]). A surface probe was used to assess the thermal characteristics of the composite samples. According to the manufacturer, the measurement uncertainty is approximately 15%. From these primary thermal properties and the known bulk density (ρ), specific heat capacity (c) and thermal effusivity (B) were calculated as follows [36]:

(1) $$c=\frac{cp\cdot 10^{6}}{\rho }$$

(2) $$B=\sqrt{\lambda \cdot c\cdot \rho }$$

Thermal effusivity quantifies a material’s ability to exchange heat with its surroundings and is influenced by both thermal conductivity and heat capacity. Measurements of λ, cp, and a using the ISOMET 2114 are well-documented in previous studies [37,38], and the underlying dynamic measurement principle is detailed by Demezhko [39]. Since both thermal parameters and bulk density are affected by moisture content, all samples were oven-dried at 110 °C to constant weight prior to testing. Bulk density was determined from the dimensions and masses of specimens used for thermal testing.

Mechanical properties were assessed using a LabTest 6.200 universal testing machine (Labortech, Opava, Czech Republic). The flexural strength was measured using a three-point bending test according to ČSN EN 1015-11 on six specimens (40 mm × 40 mm × 160 mm) at a loading rate of 0.1 MPa (~0.25 mm/min) [40]. Compressive strength was determined according to ČSN ISO 1920-10 on eight fractured halves of the flexural specimens (40 mm × 40 mm × 40 mm) using contact plates at a loading rate of 0.5 MPa/s (~0.25 mm/min) [41]. Tests were conducted 7 days after accelerated curing. Mechanical properties were measured both at ambient conditions (without thermal exposure) and after subjecting the samples to high-temperature treatment followed by cooling. Heating was carried out in a high-temperature furnace at 800 °C and 1100 °C with a ramp rate of 5 °C/min and a dwell time of 1 h. The following two cooling methods were employed: slow cooling inside the furnace (5 °C/min) and rapid quenching in water (shock cooling). Prior to mechanical testing, shock-cooled samples were oven-dried at 120 °C for 24 h. For each material variant, six specimens were tested for flexural strength.

The distribution of fillers within the composites was investigated using a Nikon Eclipse LV150N optical microscope (Nikon Metrology NV, Tokyo, Japan) in reflected light mode. Small cubic samples (10 mm × 10 mm × 10 mm) were prepared by excising them from larger blocks of the composite materials, which were originally intended for thermal property measurements. Care was taken to select areas within the blocks that were free of obvious defects, pores, and air bubbles for the preparation of these microstructural analysis samples. The excised cubic samples were then embedded in dental acrylic within cylindrical molds commonly used for metallographic sample preparation. After demolding, the samples underwent coarse grinding and subsequent polishing. However, due to the inherent hardness of the chamotte and binder particles, fine polishing without the introduction of surface scratches proved unfeasible. Observations were conducted using a TU Plan Fluor BD (Nikon Metrology NV, Tokyo, Japan) 5× objective and 10× eyepieces, resulting in a total magnification of 50×. Images were captured with a DFK 33UX250 digital camera (Nikon Metrology NV, Tokyo, Japan).

### 2.3. Preparation of Composite Material

The thermal energy storage geopolymer composite was developed using a geopolymer binder, a thermostable filler, and a metallic thermal energy storage filler. The geopolymer binder was selected based on our previous research and experimental results [21,42,43], which demonstrated its low viscosity and favorable mechanical performance (dynamic viscosity: 0.653 Pa·s; compressive strength: 36.7 MPa after 7 days of curing).

To prepare the binder, an alkaline activator, the aluminosilicate precursor Mefisto L_05_, and distilled water were combined. The alkaline activator was formulated from commercially available potassium waterglass and potassium hydroxide so that the K:Si molar ratio was 2.1. The alkaline activator was prepared 24 h in advance to ensure complete dissolution and reactivity. The binder was then synthesized by mixing the pre-dried aluminosilicate raw material (dried at 120 °C for 24 h) with the alkaline activator in a planetary mixer (Kenwood Chef XL KVL4100S, Kenwood Ltd., Havant, UK) (the mixer speed was approx. 120 rpm) at ambient laboratory temperature for 5 min. Distilled water was subsequently added to achieve the target water content, followed by another 5 min of mixing. The final binder contained 30 wt.% total water, accounting for both the water added during homogenization and any free water present in the raw materials. The molar ratios of the key constituents in the binder were carefully controlled to ensure optimal geopolymerization. The Me/Al molar ratio (where Me = K + Na) was maintained at 1.0, the Si/Al ratio at 1.5, and the Ca/Si and Me/Si ratios at 0.002 and 0.67, respectively.

Once the geopolymer binder was prepared, the thermostable filler (ground chamotte) was added in the following three different weight ratios relative to the binder: 20%, 30%, and 40% (corresponding to binder-to-filler ratios of 80:20, 70:30, and 60:40, respectively). This mixture was blended in the planetary mixer for an additional 3 min.

Next, the thermal energy storage filler—waste steel chips—was incorporated into the geopolymer mixture (i.e., binder + chamotte) in varying proportions of 0, 10, 20, 30, and 40 wt.% relative to the total composite mass. The resulting composite mixtures were mixed for another 3 min to ensure uniform dispersion of the steel filler.

The fresh composites were poured into silicone molds and subjected to vibration on a vibrating table (VSB-40, BRIO Hranice s. r.o., Hranice, Czech Republic) for 3 min to remove the maximum amount of entrapped air. The molds were then sealed in HDPE bags and cured in a laboratory oven (UF110, Memmert GmbH + Co. KG, Schwabach, DE) manufacture/company, city, abbreviated state (for USA/Canada), country) at a constant 60 °C for 4 h. After curing, the samples were demolded and allowed to mature for 7 days at ambient laboratory temperature (22 °C) while wrapped in bags. This curing protocol was adapted from the procedure reported by Rovnaník [22], ensuring complete setting and stable mechanical and thermal performance of the composites. Manipulating steel chips necessitates strict adherence to safety protocols to safeguard workers’ health. Personal protective equipment for eye and hand protection is fundamental, as is appropriate protective clothing. Furthermore, potential chemical contamination of the steel waste must be considered.

The naming convention for the prepared samples was designed to clearly reflect their composition. Each sample name consists of the initials G (geopolymer binder), C (chamotte), and W (waste steel), followed by the mass percentage of each component. The mass contents of the binder and chamotte are expressed relative to the geopolymer mixture (binder + chamotte), while the steel content is relative to the overall composite. Table 4 presents the designations of the prepared composite materials with varying filler contents.

## 3. Results and Discussion

### 3.1. Thermal Properties—Thermal Effusivity

**Figure 5:**
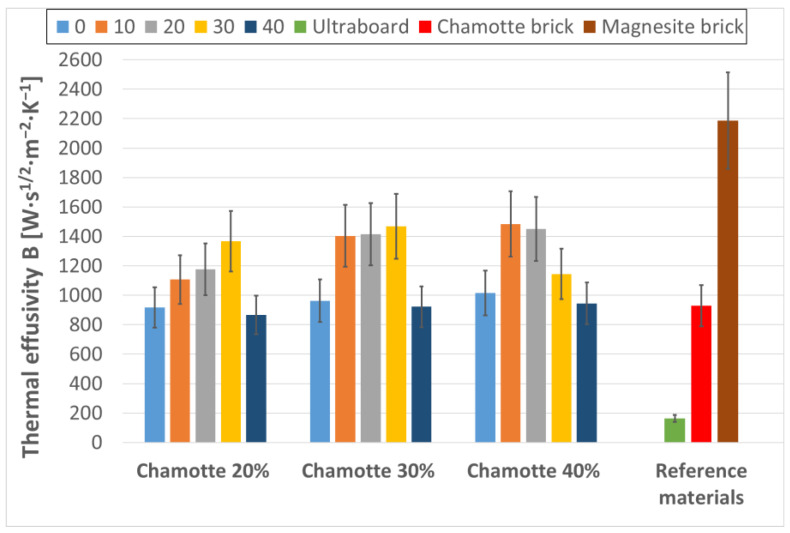
Thermal effusivity of the prepared geopolymer composites with 0, 10, 20, 30 and 40 wt.% waste steel, alongside reference materials: Ultraboard, chamotte brick, and magnetite brick.

The thermal properties of the developed thermal energy storage geopolymer composites—including bulk density and thermal effusivity—are summarized in Table 5. For comparative purposes, the corresponding properties of selected reference materials are presented in Table 6. Figure 5 presents the thermal effusivity of both the prepared composite materials and the reference materials.

As shown in the data, the bulk density of the composites strongly influenced their thermal effusivity. In general, increasing the proportion of the thermostable filler (chamotte) improved thermal properties due to the associated increase in bulk density [44]. However, this effect was limited at higher filler contents. Moreover, when large amounts of waste steel were incorporated, the viscosity of the geopolymer matrix increased, which hindered proper wetting of the metallic filler particles. This likely led to the formation of interfacial voids and increased porosity, which could have reduced the overall bulk density of the composite. This behavior has also been reported in the previous study [45], highlighting the critical role of workability and filler dispersion in maintaining composite integrity. For composites containing 20% and 30% chamotte in the geopolymer mixture, the highest bulk densities were achieved in samples G80C20W20, G80C20W30, G70C30W20, and G70C30W30. For those with 40% chamotte, the densest samples were G60C40W10 and G60C40W20. In contrast, samples containing 40 wt.% waste steel (G80C20W40, G70C30W40, and G60C40W40) consistently exhibited reduced bulk densities, likely due to increased air entrapment [45]. This reduction in bulk density also applied to one sample with 30 wt.% waste steel and 40 wt.% chamotte in the geopolymer mixture (G60C40W30).

Thermal effusivity showed a strong positive correlation (r = 0.998) with bulk density across all samples, with maximum values aligning with the densest composites. Interestingly, the highest specific mass heat capacities were recorded for samples with either no waste steel or high waste steel content, specifically G80C20W00, G80C20W40, G70C30W40, and G60C40W40, possibly due to compositional effects influencing the microstructure and energy storage capacity of the composites [45]. This seemingly nonlinear trend can be attributed to different microstructural mechanisms. For samples without waste steel (0 wt.%) the high specific mass heat capacity is likely due to the optimal geopolymerization and density of the pure geopolymer–fireclay matrix, which allows for efficient heat storage within its structure. In contrast, for samples with a high waste steel content (40 wt.%) there is likely a reduction in bulk density due to increased air entrapment, but the inherently high specific mass heat capacity of the steel itself contributes significantly to the overall mass-based heat storage capacity. Furthermore, at very high filler contents, subtle changes in interfacial bonding and the potential formation of fine micropores at the geopolymer–steel interface, which are not readily visible by the optical microscopy used, may affect the localized heat distribution and lead to an apparent increase in the specific mass heat capacity by creating ‘heat traps’ [45]. Among all evaluated thermal parameters, thermal effusivity (B) is the most comprehensive indicator of a material’s capacity for thermal energy exchange [46]. The highest thermal effusivity value (1484 W·s^1/2^·m^−2^·K^−1^) was observed for sample G60C40W10, and other composites such as G70C30W10, G70C30W20, G70C30W30, and G60C40W20 also demonstrated elevated effusivity.

Compared to reference materials, the thermal effusivity of the optimized geopolymer composites exceeded that of chamotte brick by more than 50%. Although magnetite brick showed higher thermal effusivity—as expected due to its greater thermal conductivity and bulk density—its industrial production is associated with significant energy consumption and high costs [47], which limits its sustainability. In contrast, the developed composites utilize low-cost, waste-derived materials, offering an economically and environmentally favorable alternative for high-performance refractory applications. It is noteworthy that thermal effusivity values for similar refractory materials are often scarce in the literature, underscoring the importance of well-characterized reference materials with outstanding thermal properties selected here as benchmarks and highlighting the novel contribution of this study in advancing thermal characterization data for sustainable composites.

### 3.2. Mechanical Properties

#### 3.2.1. Flexural Strength

**Figure 6:**
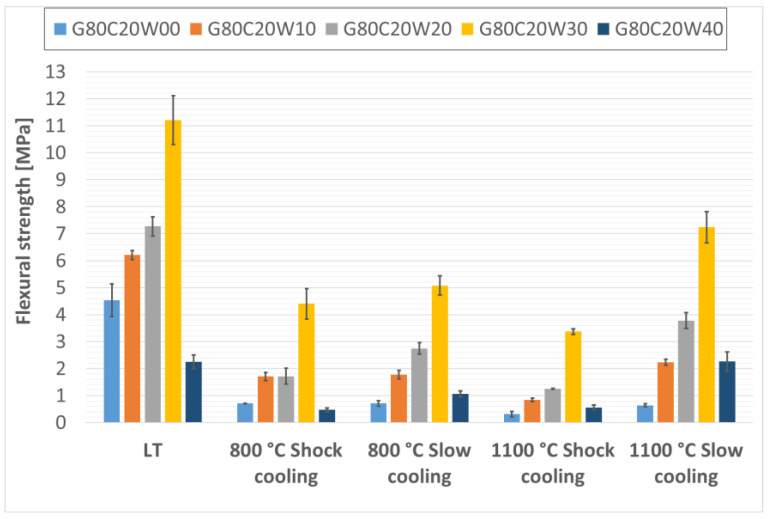
Flexural strengths of samples containing 20% particulate chamotte in the geopolymer mixture.

**Figure 7:**
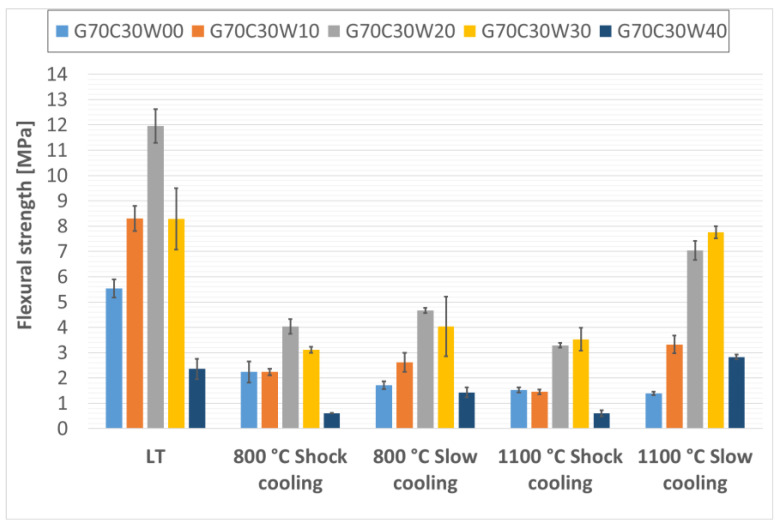
Flexural strengths of samples containing 30% particulate chamotte in the geopolymer mixture.

**Figure 8:**
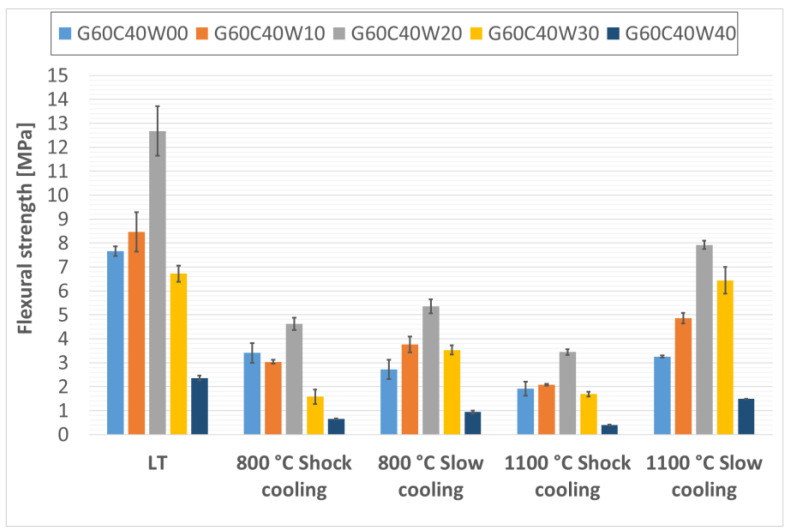
Flexural strengths of samples containing 40% particulate chamotte in the geopolymer mixture.

The flexural strengths of the prepared thermal energy storage composite materials, both before and after thermal exposure, are presented in Figure 6, Figure 7 and Figure 8. Analysis of the data presented in these figures (Figure 6, Figure 7 and Figure 8) reveals a clear positive impact of incorporating waste steel on the flexural strength of the composites. This enhancement aligns with findings reported by Gao, Gailitis, Rashad, and Zhang in their respective studies [48,49,50,51]. The improved flexural strength is attributed to the ductile nature of the steel chips, which act as effective crack bridging elements, redistributing stress and inhibiting crack propagation under bending loads. This bridging effect enhances the material’s resistance to tensile stresses developed during flexure, thereby increasing the energy required for fracture.

For composites with 20% chamotte particles, the highest flexural strength was achieved with a 30% addition of waste steel (sample G80C20W30), reaching 11.21 MPa, consistently both before and after thermal exposure.

In contrast, for composites containing 30% and 40% chamotte, the peak flexural strengths were observed in samples with 20% waste steel subjected to controlled cooling (samples G70C30W20 and G60C40W20), with strengths of 11.96 MPa and 12.68 MPa, respectively.

This shift in maximum flexural strength toward samples with lower waste steel content at higher chamotte percentages likely reflects the overall structural integrity of the specimens. Higher total filler content tends to introduce more air voids and other defects, which can weaken the material [48,49,50,51].

Furthermore, rapid cooling (shock cooling) resulted in reduced flexural strengths across all samples compared to those cooled under controlled conditions. This decline was noticeable in specimens exposed to 800 °C and became more pronounced at 1100 °C. A similar phenomenon was reported by Rovnaník in metakaolin-based geopolymer mortars [52]. The reduction in strength is likely attributable to alterations in the phase composition of the geopolymer binder and modifications to the surface characteristics of the waste steel particles. Specifically, exposure to elevated temperatures induces significant phase changes in the geopolymer matrix. As observed in a previous study on metakaolinite-based geopolymers [21], the amorphous geopolymer phase can transform into crystalline phases. While such crystallization can lead to densification and increased hardness, it is also associated with significant shrinkage of the geopolymer matrix and the development of internal stresses due to differential thermal expansion between the newly formed crystalline phases, the residual amorphous matrix, and the fillers. Rapid cooling exacerbates these thermal stresses, leading to increased microcracking and weakening of the interfacial bond, which is detrimental to flexural strength. Notably, the effect of rapid cooling on the machining waste steel structure and properties was minimal, likely due to the predominance of low-carbon steels in the machining waste.

#### 3.2.2. Compressive Strength

**Figure 9:**
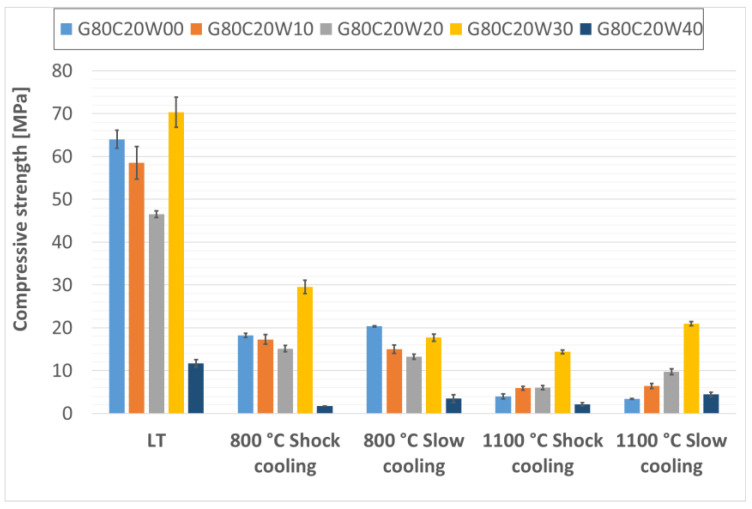
Compressive strength of samples containing 20% particulate chamotte in the geopolymer matrix.

**Figure 10:**
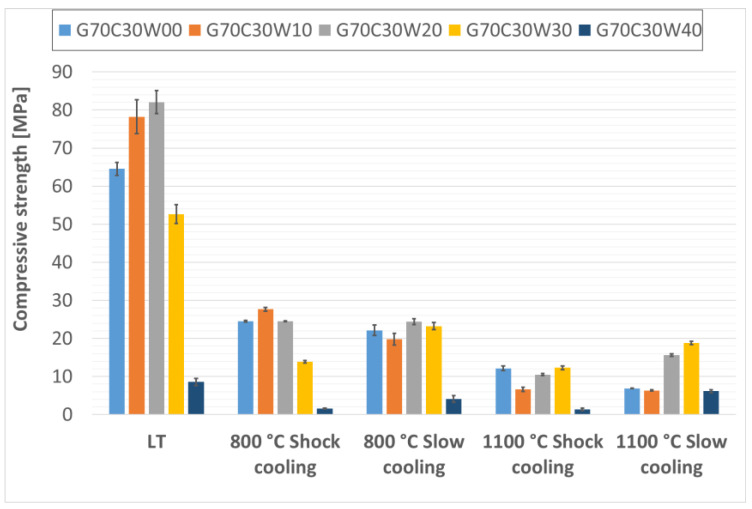
Compressive strength of samples containing 30% particulate chamotte in the geopolymer matrix.

**Figure 11:**
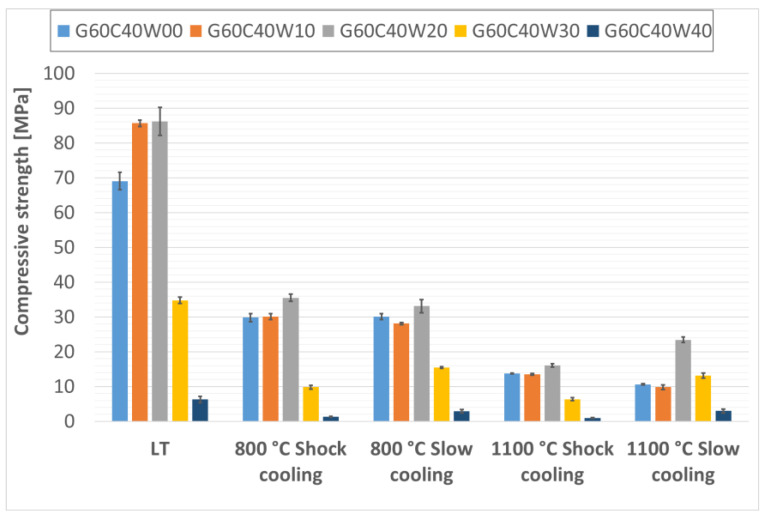
Compressive strength of samples containing 40% particulate chamotte in the geopolymer matrix.

The compressive strengths of the fabricated thermal energy storage composite materials are presented in Figure 9, Figure 10 and Figure 11. While the addition of steel waste from cutting processes improved the compressive strength of the geopolymer composites, the effect was less pronounced than for flexural strength. This is likely due to the morphology of the steel waste, which consists of elongated particles that serve more effectively as reinforcement under bending stresses rather than compressive loads. Under compressive loading, the primary failure mechanism is often associated with the propagation of microcracks from existing flaws or pores within the matrix. While steel chips can contribute to load distribution, their elongated geometry is less effective in arresting these cracks under purely compressive forces compared to their role in bridging tensile cracks during flexure. Therefore, the inherent compressive strength of the geopolymer matrix and the presence of any voids become more dominant factors. Similar findings have been reported by Gao, Gailitis, Rashad, and Zhang in their respective studies [48,49,50,51].

In this study, the highest compressive strengths were observed in samples that had not been subjected to thermal exposure. As with the flexural strength, the compressive strength was highest for the composite material with the lowest geopolymer viscosity, specifically, for the sample containing 20% fireclay and 30% waste steel (G80C20W30), which reached 70.32 MPa before and after thermal exposure.

For composites containing 30% and 40% chamotte, the highest compressive strengths were recorded in samples with 20% steel waste after exposure to low temperature (LT) conditions; G70C30W20 reached 82.07 MPa, and G60C40W20 achieved 86.18 MPa.

Among the thermally treated samples, the beneficial effect of controlled cooling on compressive strength was noticeably less than what was observed for flexural strength, consistent with findings reported by Rovnaník [52]. This again may be attributed to the geometry of the steel chips, whose elongated form offers greater reinforcement under tensile (bending) stresses than compressive ones.

Nevertheless, even after exposure to a high temperature of 1100 °C, the composite samples maintained compressive strengths around 20 MPa, surpassing those typically found in conventional concrete under similar thermal conditions [53,54]. The relatively low compressive strength observed in samples with the highest steel content (40% by total composite weight) is likely due to an increased number of pores and air voids within the specimens, which can compromise mechanical performance. The slight increase in compressive strength observed in some samples after exposure to 1100 °C can be attributed to viscous sintering of the geopolymer matrix and the formation of new, stiffer crystalline phases, which lead to densification and improved load-bearing capacity. However, the overall reduction in strength at higher temperatures, particularly after shock cooling, is indicative of the cumulative damage from thermal stresses and significant microcracking caused by the mismatch in thermal expansion coefficients between the geopolymer and the steel/chamotte fillers, as well as by the volumetric changes associated with crystallization.

#### 3.2.3. Thermal Effusivity vs. Flexural and Compressive Strength

**Figure 12:**
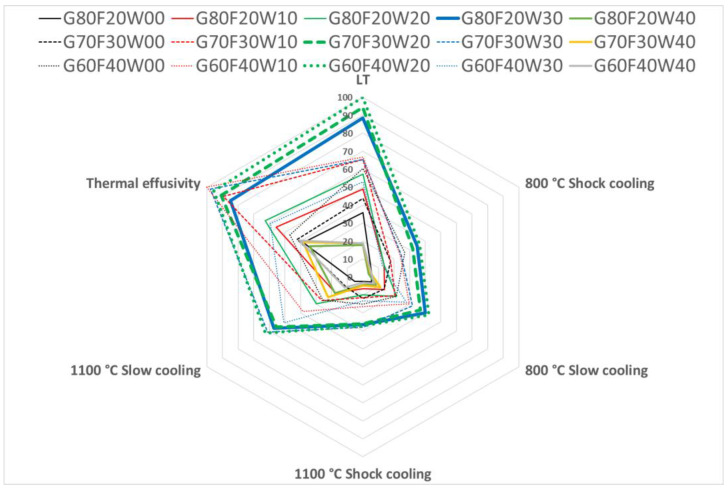
Flexural strength and thermal effusivity of composite materials incorporating waste steel.

**Figure 13:**
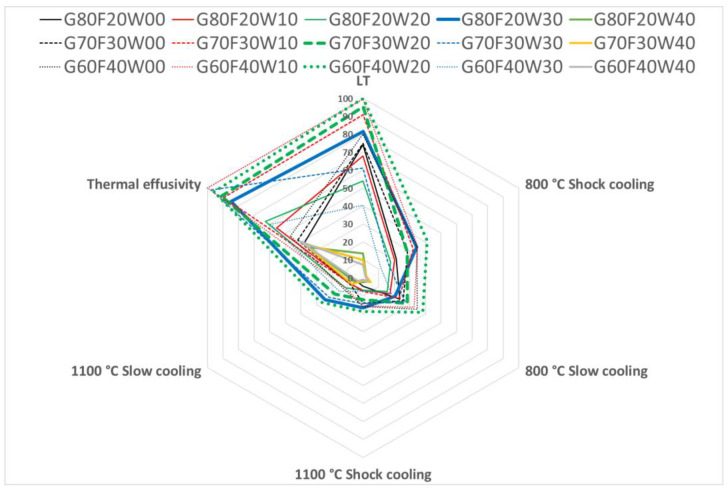
Compressive strength and thermal effusivity of composite materials incorporating waste steel.

To support the practical selection of the most appropriate material for specific applications, network graphs were developed to simultaneously visualize all evaluated properties (Figure 12 and Figure 13). These graphical representations enable a comparative assessment of geopolymer composite formulations based on key performance indicators relevant to intended uses.

In constructing these graphs, values for flexural strength, compressive strength, and thermal effusivity were normalized and expressed as percentages, with the maximum value in each category set to 100%. This approach facilitates a direct comparison across different formulations, making it easier to identify the most balanced and effective material composition for specific requirements.

Among the tested materials, the composites G80C20W30, G70C30W20, and G60C40W20 demonstrated the most favorable combination of high thermal effusivity, with strong mechanical performance in both flexural and compressive strength. As such, these formulations were identified as the most versatile candidates, offering a wide range of application potential in thermal energy storage and structural applications.

#### 3.2.4. Structure and Particle Distribution in the Composite

**Figure 14:**
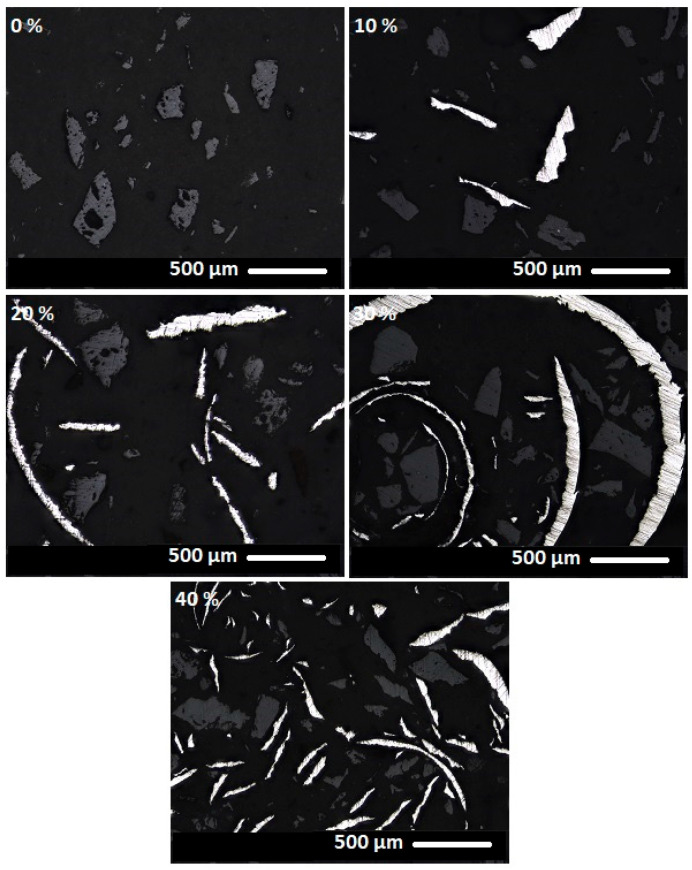
Composite materials with a binder-to-chamotte ratio of 80:20 and steel chip mass concentrations of 0, 10, 20, 30 and 40 wt.%.

**Figure 15:**
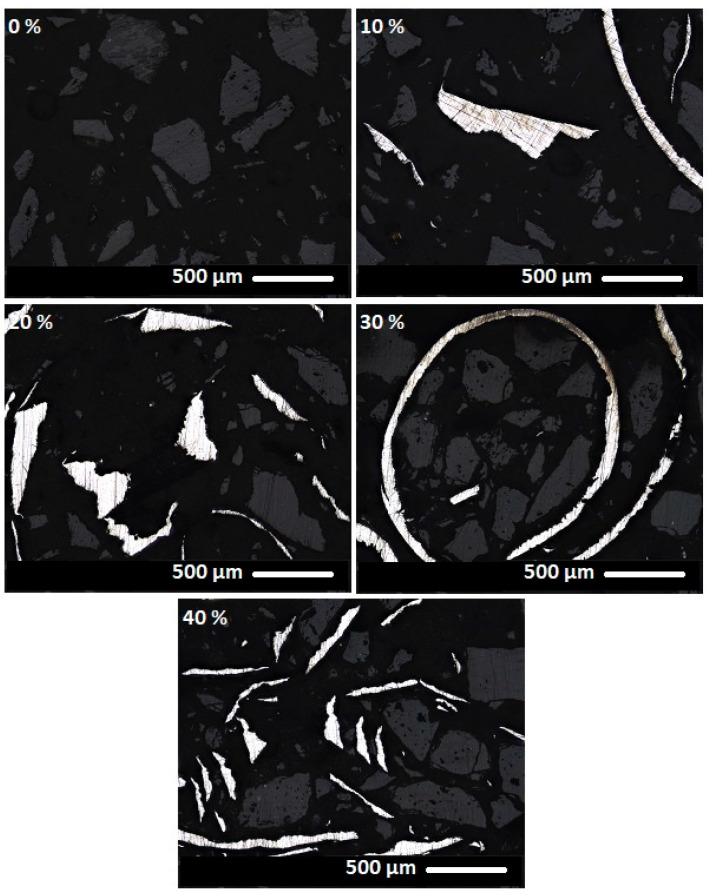
Composite materials with a binder-to-chamotte ratio of 70:30 and steel chip mass concentrations of 0, 10, 20, 30 and 40 wt.%.

**Figure 16:**
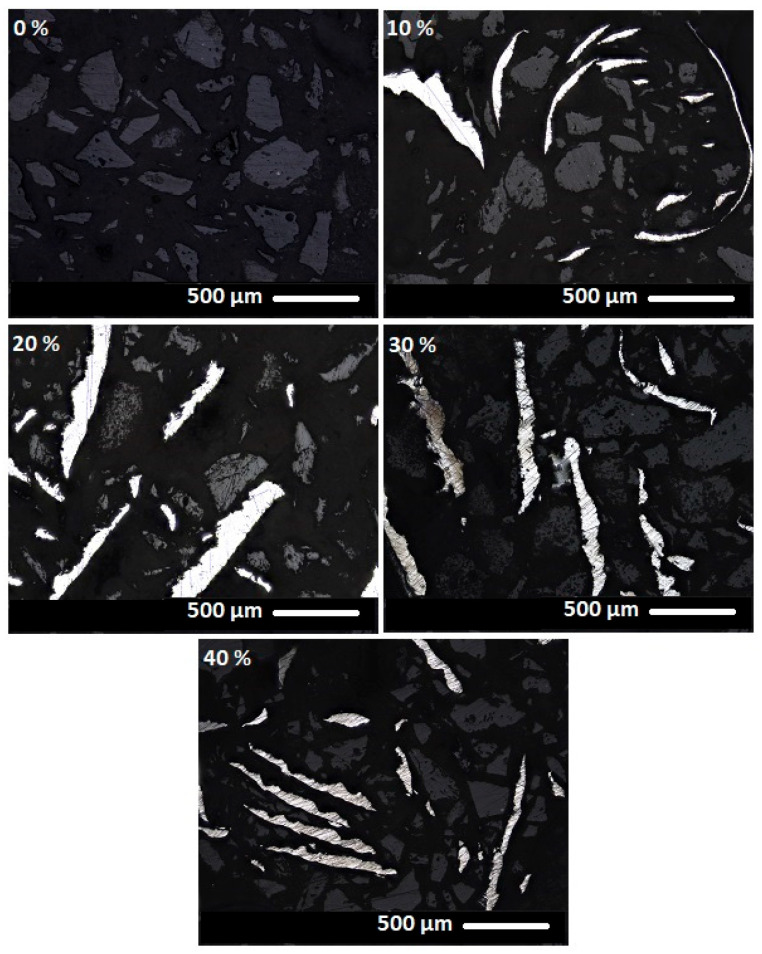
Composite materials with a binder-to-chamotte ratio of 60:40 and steel chip mass concentrations of 0, 10, 20, 30 and 40 wt.%.

Figure 14, Figure 15 and Figure 16 present images of geopolymer composites prepared with varying binder-to-chamotte ratios and different steel chip contents.

The microstructural properties of geopolymer composites are clearly discernible in the images. The darkest regions correspond to the geopolymer binder, while lighter areas represent the thermostable chamotte filler. The brightest regions indicate the distribution of waste steel chips. The steel chips exhibit a wide range of shapes yet appear to be well-integrated into the matrix, maintaining good contact with the surrounding binder at the observed scale. Similarly, the geopolymer binder demonstrates effective adhesion to the chamotte particles at the observed scale. The images reveal no signs of porosity, suggesting good material homogeneity at the observed scale. However, it is important to note that these visual observations were conducted on selected, relatively small areas of the sample’s cross-section and did not capture potential broader heterogeneities.

## 4. Conclusions

This study successfully investigated the development and characterization of metakaolinite-based geopolymer composites, incorporating thermally stable chamotte and waste steel chips for enhanced thermal energy storage capabilities. The comprehensive evaluation of the thermal and mechanical performance of these materials was evaluated both before and after exposure at 800 °C and 1100 °C, under varying cooling regimes. Based on the experimental findings, the following conclusions can be drawn:

- Optimized Thermal Properties through Density Control: Bulk density was identified as a critical factor governing the thermal properties of the composites. Higher bulk densities consistently correlated with superior thermal storage capabilities. Importantly, the inclusion of chamotte also positively contributed to thermal performance. The highest thermal effusivity was observed in the following two formulations: one containing 40% chamotte and 10% waste steel (G60C40W10, 1484 W·s^1/2^·m^−2^·K^−1^), and another with 30% chamotte and 30% waste steel (G70C30W30, 1467 W·s^1/2^·m^−2^·K^−1^). These values represent a significant improvement of over 50% compared to conventional chamotte brick, positioning these composites as highly competitive sustainable alternatives for thermal energy storage applications.
- Enhanced Mechanical Resilience with Waste Steel Inclusion: The incorporation of waste steel chips demonstrably improved the mechanical properties of the geopolymer composites. This enhancement was particularly pronounced in flexural strength due to the crack-bridging effect of the elongated steel particles. Controlled cooling after thermal exposure, especially at 1100 °C, further benefited flexural strength. The optimal mechanical performance was achieved by sample G60C40W20 (40% chamotte, 20% waste steel), which exhibited the highest flexural strength of 12.68 MPa and the highest compressive strength of 86.18 MPa under low-temperature (LT) conditions. This highlights the potential of waste steel as a valuable reinforcing filler in geopolymer systems.
- Material Selection via Network Graphs: The application of network graphs proved to be an invaluable tool for the assessment and selection of optimal composite formulations. By simultaneously visualizing flexural strength, compressive strength, and thermal effusivity, these graphs enabled a comprehensive comparative analysis. Based on this multi-criteria evaluation, the formulations G80C20W30, G70C30W20, and G60C40W20 were identified as the most promising materials, offering a versatile balance of robust mechanical performance and efficient thermal energy exchange. This methodology can be broadly applied for tailoring composite materials to specific application demands.
- Structural Integrity and Compatibility: To prepare samples for microstructural analysis, areas within the blocks that were free from obvious defects, pores, and air bubbles were selected. Optical microscopy subsequently confirmed good compatibility among the geopolymer binder, chamotte particles, and waste steel chips. The observed homogeneity and lack of significant porosity at the examined scale suggest a well-integrated composite matrix. While further high-resolution imaging techniques could elucidate finer interfacial details, these findings support the robust nature of the developed composites.

In conclusion, this research successfully demonstrates the viability of utilizing waste metallic materials in metakaolinite-based geopolymers to produce multifunctional composites with enhanced thermal energy storage and mechanical properties. The findings emphasize that a tailored approach to composite formulation, guided by comprehensive evaluation tools like network graphs, is crucial for optimizing material performance for specific industrial and energy storage applications. This work contributes significantly to the development of sustainable and high-performance materials from industrial waste streams.

## Figures and Tables

**Table 1:** Chemical composition (wt.%) of raw materials.

Raw Materials	Material Composition [wt.%]														
	LOI_a_	H_2_O	SiO_2_	Al_2_O_3_	TiO_2_	Fe_2_O_3_	K_2_O	Na_2_O	CaO	MgO	P_2_O_5_	V_2_O_5_	Cr_2_O_3_	ZrO_2_	SrO
Mefisto L_05_	1.53	0.34	51.90	42.50	1.66	0.88	0.83	0.04	0.16	0.16	0.07	0.05	0.03	0.02	0.01
Potassium silicate	-	60.44	27.00	0.05	-	-	12.12	0.38	-	-	-	-	-	-	-
Chamotte	1.48	1.03	53.71	39.82	2.05	1.26	0.96	0.03	0.20	0.13	0.07	0.06	0.03	0.04	0.02

^a^ LOI = Loss on ignition.

**Table 2:** Physical properties of the raw materials and chamotte.

Material	Specific Gravity [kg/m^3^]	Bulk Density [kg/m^3^]	Particle Size		Specific Surface Area (BET) [m^2^/g]
			D_50_ [µm]	D_90_ [µm]	
Mefisto L_05_	2573	541	3.73	10.55	15.6
Chamotte	2741	1428	389.44	720.13	1.8

**Table 3:** Chemical composition (wt.%) of reference materials.

Reference Materials	Material Composition [wt.%]														
	LOI_a_	H_2_O	SiO_2_	Al_2_O_3_	TiO_2_	Fe_2_O_3_	K_2_O	Na_2_O	CaO	MgO	P_2_O_5_	V_2_O_5_	Cr_2_O_3_	ZrO_2_	SrO
Ultraboard	0.04	0.41	32.10	67.60	-	0.11	-	0.14	0.04	-	-	-	-	-	-
Chamotte brick	0.31	0.19	53.90	38.90	1.29	2.13	2.57	0.27	0.27	0.53	0.06	-	0.02	0.04	0.01
Magnetite brick	0.00	0.16	16.00	3.42	0.49	74.50	0.36	0.56	-	1.62	0.67	0.11	0.02	-	-

^a^ LOI = Loss on ignition.

**Table 4:** Marking of prepared composite materials with different weight contents of fillers.

Composite Marking	Ratio of Geopolymer Binder and Chamotte in the Geo-Polymer Mixture	Steel Content in Composite Material [wt.%]
G80C20W00	80:20	0
G80C20W10		10
G80C20W20		20
G80C20W30		30
G80C20W40		40
G70C30W00	70:30	0
G70C30W10		10
G70C30W20		20
G70C30W30		30
G70C30W40		40
G60C40W00	60:40	0
G60C40W10		10
G60C40W20		20
G60C40W30		30
G60C40W40		40

**Table 5:** Bulk density and thermal properties of the geopolymer composite materials.

Samples		Bulk Density ρ [kg·m^−3^]	Thermal Conductivity Coefficient λ [W·m^−1^·K^−1^]	Specific Vol. Heat Capacity cp [J·m^−3^·K^−1^]	Temperature Diffusivity a [m^2^·s^−1^]	Specific Mass Heat Capacity c [J·kg^−1^·K^−1^]	Thermal Effusivity B [W·s^1/2^·m^−2^·K^−1^]
Chamotte 20%	G80C20W00	1466	0.50	1.68	0.30	1143	917
	G80C20W10	1763	0.76	1.62	0.47	918	1107
	G80C20W20	1828	0.84	1.64	0.56	897	1176
	G80C20W30	1876	1.13	1.66	0.68	885	1367
	G80C20W40	1335	0.50	1.51	0.33	1129	867
Chamotte 30%	G70C30W00	1736	0.55	1.68	0.33	968	963
	G70C30W10	1878	1.15	1.72	0.67	917	1404
	G70C30W20	2082	1.23	1.63	0.75	784	1415
	G70C30W30	2201	1.36	1.59	0.86	721	1467
	G70C30W40	1239	0.57	1.50	0.38	1213	922
Chamotte 40%	G60C40W00	1831	0.64	1.62	0.39	883	1015
	G60C40W10	1999	1.18	1.87	0.63	936	1484
	G60C40W20	2119	1.27	1.67	0.76	786	1452
	G60C40W30	1675	0.80	1.64	0.48	981	1144
	G60C40W40	1458	0.61	1.47	0.41	1010	945

**Table 6:** Bulk density and thermal properties of the reference materials.

Samples	Bulk Density ρ [kg·m^−3^]	Thermal Conductivity Coefficient λ [W·m^−1^·K^−1^]	Specific Vol. Heat Capacity cp [J·m^−3^·K^−1^]	Temperature Diffusivity a [m^2^·s^−1^]	Specific Mass Heat Capacity c [J·kg^−1^·K^−1^]	Thermal Effusivity B [W·s^1/2^·m^−2^·K^−1^]
Ultraboard	400	0.08	0.08	0.31	786	162
Chamotte brick	1951	0.60	0.60	1.44	736	930
Magnetite brick	3818	2.76	2.76	1.73	453	2186

## Data Availability

The original contributions presented in this study are included in the article. Further inquiries can be directed to the corresponding authors.
